# Australian women’s experiences of post-partum rectus diastasis: A qualitative study

**DOI:** 10.1177/17455057241233123

**Published:** 2024-04-05

**Authors:** Siobhan Elizabeth Fitzpatrick, Kristen Foley, Tamara Crittenden, David Watson, Nicola R Dean

**Affiliations:** 1 College of Medicine and Public Health, Flinders University, Adelaide, SA, Australia; 2 Department of Plastic and Reconstructive Surgery, Flinders Medical Centre, Adelaide, SA, Australia; 3 Centre for Public Health, Equity and Human Flourishing, Torrens University, Adelaide, SA, Australia; 4 Department of Surgery, Flinders Medical Centre, Adelaide, SA, Australia

**Keywords:** abdominoplasty, qualitative, rectus diastasis, surgery

## Abstract

**Background:**

Post-partum rectus diastasis, or the separation of the abdominal muscles after pregnancy, occurs in conjunction with physical symptoms and impaired quality of life. In Australia, health funding for surgery to treat diastasis was ceased in 2016, but reinstated in mid-2022, providing a unique context from which women’s experiences of this condition can be analysed.

**Objectives:**

The objective is to examine the experiences of Australian women with post-partum rectus diastasis.

**Design:**

This is an interview-style study with qualitative content analysis.

**Methods:**

Women diagnosed with rectus diastasis were recruited to complete a baseline questionnaire (n = 45). Twenty-three responded to invitation for one-on-one interview via Zoom® between November 2021 and May 2022. Interviews were recorded, transcribed, and analysed using qualitative content analysis to identify key themes.

**Results:**

Eighteen women had undergone caesarean section and eight had twins. Thirteen had private health insurance. Women were most often diagnosed by a physiotherapist (n = 10). Key themes identified included changed physical appearance and function; issues with self-esteem and intimacy; barriers to treatment; lack of recognition as a medical condition; and overall frustration. The impact of rectus diastasis extended beyond physical and psychological symptoms to affect women’s social functioning, child rearing, and return to work. There was a complex interaction between healthcare providers’ knowledge of rectus the removal of funding for surgical treatment, and limitations of conservative therapy, with women’s lived experiences and symptoms. The lack of an established medical definition also influenced the experiences of these women and their engagement with treatment.

**Conclusion:**

This study contextualizes women’s experience of post-partum rectus diastasis with respect to the unique landscape of Australia’s healthcare economy and provides evidence of women’s absorption of health policy surrounding this condition. Our qualitative analysis provides critical knowledge for future quantitative studies, the results of which in combination could advance the definition of rectus diastasis and inform healthcare policy surrounding treatment.

## Introduction

Post-partum rectus diastasis entails the separation of the rectus abdominis muscles following pregnancy.^1^ It is reported to be associated with symptoms such as back pain, urinary incontinence, abdominal wall weakness, and reduced quality of life.^2–6^ In Australia, if a woman develops post-partum rectus diastasis after pregnancy, conservative management can be accessed via private healthcare from physiotherapists with or without an insurance rebate. If women require surgical repair of rectus diastasis, it is typically performed as part of an open abdominoplasty. In 2016, public funding and private insurance coverage for abdominoplasty surgery in Australia was restricted to exclude women seeking surgery for pregnancy-related issues such as rectus diastasis, leaving women to self-fund the surgery as a cosmetic procedure in the private hospital system, often costing tens of thousands of dollars out of pocket.^7^ After a community-led petition in 2020 to restore funding, along with recently published quantitative health-related quality of life research,^5,6,8^ a new item number for abdominoplasty for post-partum rectus diastasis was implemented in 2022 (item no. 30175).^9^

In the medical literature, rectus diastasis that persists in women years after pregnancy remains poorly defined^10^ and is consequently poorly understood by healthcare professionals.^11^ To date, scholarly understanding has been drawn predominantly from quantitative research using dynamometers, functional assessment tools, and validated questionnaires such as patient-reported outcome measures (PROMs). There is a paucity of qualitative research about women’s experiences of rectus diastasis as well as a lack of knowledge about the impacts the condition has on women.^12^ Eriksson et al.’s^13^ in-depth interview study of 19 women and Vicente-Campos et al.’s^14^ analysis of 108 responses from Spanish women to two open-ended survey questions, both conveyed women feeling misunderstood by those around them or during treatment as well as within the health system and by healthcare professionals specifically.^13,14^ Both groups advocated for further in-depth qualitative research about women’s experiences of this condition.

Our qualitative study responds to this call and aims to help situate women’s experiences in relation to different healthcare systems and cultural contexts replete with political and gendered dimensions. Given that access to surgical repair differs widely between health systems^15–17^ and that abdominoplasty for post-partum rectus diastasis in Australia was recently removed and then re-introduced through public funding, the Australian context of this study provides a rich terrain for understanding women’s experiences of the condition and its associated symptoms in relation to their daily lives, inter-personal relationships, engagements with healthcare, and society in general. The aim of this study is therefore to elucidate the experiences of Australian women living with post-partum rectus diastasis. These qualitative findings will in turn inform quantitative studies to advance the development of a definition and understanding of rectus diastasis that is useful to policymakers, fiscal experts, medical practitioners, and women who live with the condition.

## Methods

### Participants

Australian women over 18 years of age, who had previously given birth and had an established diagnosis of post-partum rectus diastasis provided by a healthcare provider or medical imaging confirmation, were eligible for this study. Women without access to a webcam or telephone, and those who did not speak English and for whom a medical interpreter was not available, were excluded. The study was advertised physically via flyers placed in Flinders Medical Centre, Flinders University, and local General Practitioner (GP) and private physiotherapy clinics in Adelaide. The study was also advertised electronically via posts in social media groups that provide support and information for women with rectus diastasis.

A snowball sampling approach was employed, with women enrolled in the study being able to share the study link electronically with other women who they considered might have rectus diastasis. The online Qualtrics survey (Qualtrics, Provo, UT, USA) included questions asking if they were diagnosed with ‘rectus diastasis’ and how this diagnosis came about, as well as demographic information such as age, body mass index (BMI), parity, singleton/multiple births, years since last pregnancy, involvement in paid work and type of work, education level, and ethnicity. Participants were invited to participate in a one-on-one interview with a female researcher with expert communication skills (S.E.F.) online via Zoom (Zoom Video Communications Inc., San José, CA, USA). Women had not previously met the interviewer but were aware that she is a medical doctor (MD) undertaking research in rectus diastasis for a doctorate and not part of their clinical care team. Women were recruited and invited for interview to develop a robust understanding of themes,^18^ as opposed to data saturation, reflecting the interconnectedness of data collection, analysis, and interpretation which is a hallmark of quality and responsive qualitative research.^19^ Ethical approval was from the Southern Adelaide Clinical Human Research Ethics Committee (approval no. 124.21).

### Sample characteristics

Forty-five women completed the questionnaire and 23 accepted invitations for one-on-one interview. The reasons for non-interview were not obtained. The average age and BMI of the women that interviewed was 38 years old and 28.4 kg/m^2^. Thirteen of the 23 women had private health insurance, 18 underwent at least one caesarean section, and 8 women gave birth to twins. Women were mostly diagnosed by a physiotherapist (n = 10), GP (n = 6), ‘other’, such as an exercise physiologist (n = 4), and plastic surgeon (n = 3). Using categories derived from the 2016 Australian Census classification for ancestry as an indication of ethnic background,^20^ women were asked to select one category that best described their ethnicity (or ‘other’ with free text), which resulted in 10 women selecting ‘Australian’, 8 ‘English’, 1 ‘Scottish’, 1 ‘Filipino’, 1 ‘Maltese’, and 2 women identifying as Aboriginal and Torres Strait Islander. Sixteen of the women interviewed were involved in paid work, and 20 had completed education beyond year 12, such as a trade, diploma, or university degree. Participant age, number of deliveries, caesarean sections, multiple births, time since last delivery, and time since diagnosis of rectus diastasis have been presented in Table 1. While the quotes and themes were explored in relation to these demographic data, no relationships observed between specific demographic factors and themes that emerged in interviews.

**Table 1.** Characteristics of participants who were interviewed.

Participant number	Age (years)	Years post-partum	Years since diagnosis	Number of deliveries	Number of caesareans	Multiples (twins)
1	37	4	2	2	1	No
2	50	1	8	4	2	No
3	27	2	2	1	1	No
4	40	5	3	2	1	Yes
5	47	6	5	2	2	No
6	36	1	1	2	2	No
7	38	4	5	4	3	Yes
8	46	7	1	1	1	No
9	38	6	5	5	5	Yes
10	35	4	5	3	3	No
11	40	11	11	4	0	No
12	36	1.5	8	4	4	No
13	46	13	19	6	0	No
14	35	0.5	4	2	2	No
15	42	19	1	2	2	No
16	27	2	2	3	0	Yes
17	40	2	2	2	2	No
18	40	2	2	5	5	Yes
19	35	4	4	1	1	Yes
20	35	3	6	2	0	No
21	33	4	5	3	0	No
22	29	2	2	1	1	Yes
23	47	1	1	2	1	Yes

### Procedure for data collection

Interviews were undertaken between November 2021 and May 2022 and followed a semi-structured approach (see Supplementary File). Questions were predominantly open-ended and asked about the women’s experience of rectus diastasis and its effects on activities of daily living, quality of life, physical health, self-perception, and sexual well-being. Women were also asked about their engagement with healthcare, sources of information used, and their overall perception of the condition. Select follow-up questions or questions to encourage expansion of answers were asked. After the first two interviews, the question order was slightly modified to facilitate participant responses. Interviews were recorded (audio or video), de-identified using a numerical identifier, and the audio recording transcribed verbatim without field notes.

Twenty-three interviews were conducted (no repeats). The interview recording times ranged from 10 to 33 min (most > 20 min). Two women interviewed had undergone abdominoplasty with muscle separation repair before enrolling in the study.

### Data analysis

Transcripts were analysed using qualitative content analysis with codes derived directly from the text, rather than being pre-formed.^21^ One researcher (S.E.F.) completed open coding and then organized these into recurring themes and subthemes. Quotes pertaining to each theme were catalogued in a Microsoft Excel spreadsheet (Microsoft Corporation, Redmond, WA, USA). A second researcher (K.F.) then co-coded the transcripts and cross-checked the themes and catalogued quotes, raising points for discussion and refinement. This process was repeated a second time to ensure an agreement was reached. There were no changes made to the initial codes; however, how the categories of quotes should be interpreted did shift during this process. The analysis and interpretation were then reviewed and agreed upon by the research team (N.R.D., T.C., and D.W.). All of these steps supported the interpretive rigour of the analysis.^22^ Transcripts and codes were not returned to participants for comment/feedback.

## Results

Inter-related themes were identified within the data and are presented here: changed physical appearance and function; issues with self-esteem and intimacy; barriers to treatment; lack of recognition as a medical condition; and overall frustration. Each of these will be elucidated here, before situating these in relationship to the social and fiscal contexts which texture understandings and treatment of post-partum rectus diastasis in Australia.

## Changed physical appearance and function

All interviewed women indicated some change in their abdominal wall following their pregnancy, which had significant impacts to their functional abilities as well as their feelings about their bodies. Half of the women described their protruding bellies gave the appearance of, and were recognized by others, as being pregnant when they were not:

I have this pouch that just sticks out. (Participant 2)

My stomach never went down . . . I’ve still got a distended stomach even now’. (Participant 5)

I look like I’m 9-months pregnant. (Participant 9)

The levels of distress associated with having a protruding belly varied among those interviewed, with some relaying the experience of ‘coning’ (where the space between the abdominal muscles specifically protrudes under tension) as disconcerting:

It literally used to look like a little alien wanting to hatch out of my stomach and it used to really freak me out. (Participant 11)

The protrusion could lead women to feel like they were living in bodies that were now ‘inside out’:

I felt like my insides were pouring out of my body. (Participant 14)

I felt like I had to kind of carry all of my internal organs to the bathroom. (Participant 22)

In addition to having a protruding belly, many women described the sensation of having no protection for their abdominal organs:

If one of my kids hits me in the stomach, it’s like they’ve just punched me literally in the gut. So yeh, it can be very painful. (Participant 18)

My 5-year-old likes playing rough and jumping on mummy, and I realise when she’s jumping on my tummy that I almost felt like she was touching my organs. (Participant 23)

In addition to these moments of vulnerability, all women interviewed reported chronic and significant back pain, even at rest:

I woke up every morning and my lower back was like someone had a knife in my lower back and that’s . . . what would wake me up in the morning. (Participant 8)

Yes, absolutely, big time. My lower back is in constant pain. (Participant 9)

Unfortunately, for me, it’s just progressively getting worse, my back is just progressively . . . I’m just in pain all the time. (Participant 16)

The back pain that some women reported, impacted heavily on their ability to care for their children:

The back pain was so significant that I couldn’t turn over in my bed, I was co-sleeping because it was easier . . . I remember getting my four-year-old, saying, ‘Can you try and put my shoes on so I can take you to Kinder’. (Participant 17)

You’d be so sore at the end of the day from lifting and not using it properly . . . I couldn’t push the pram with all the kids in it at one point. (Participant 10)

One of the women described getting relief for her back pain using an abdominal binder:

I get a lot of back pain . . . my whole stomach [has] no support. So I wear [the binder] and it does reduce my back pain. (Participant 11)

The two women who underwent surgery described a great physical improvement, particularly with regards to back pain, characterized by the following quote:

I’ve had it all repaired now, and the difference it’s made. I haven’t had a sore back at all since the day it was done. (Participant 20)

When prompted about urinary incontinence, seventeen women reported issues, although it was not universally reported, making it unclear if it is specific to having diastasis:

I usually get . . . stress incontinence . . . when I’m laughing or it’s difficult sometimes to hold urine. (Participant 6)

Absolutely, yes, I peed my pants the other night. Embarrassing, but my bladder just constantly gets weaker. (Participant 9)

I get more worried about if I’m going to leak too much that this is going to be an issue . . . carrying a spare pair of pants in my car, just in case you know like if I sneeze too hard or I’ve gone for a run and that’s it, it’s all over. (Participant 11)

Yes, if I laugh or sneeze. If I laugh I pee myself. I can’t jump, if I jump I pee myself. So the kids are like, ‘jump on the trampoline, Mum’ I’m like. ‘I can’t I’ll wee myself’. (Participant 16)

All of the women expressed that rectus diastasis impacted on their paid work or their ability to care for their children:

No, but as a physio it’s very physical work. It’s quite physical getting people up and about out of bed, so that has meant I’ve had to be super hypervigilant and aware of my own manual handling and keeping myself safe. (Participant 4)

I’m an agricultural scientist and I used to work out in paddocks a lot and now I do a lot more desk work . . . I don’t trust my body any more to be able to do what it used to do. (Participant 19)

I’m a PE teacher, and I couldn’t teach my classes properly because I was always afraid of moving the wrong way, hurting my back or having it lock. (Participant 20)

I’m a stay-at-home mum. It affects things around the house. Picking kids up has always been more of a challenge. My back hurts. (Participant 21)

I’m a nurse and it’s quite a physical job . . . There are still certain movements like pushing beds and things that I’m really nervous about doing . . . I didn’t really return to fulltime work. I tried to go back fulltime, and it just started to hurt so I just left it alone for a little bit. (Participant 22)

I live by myself . . . I work all day at home, I struggle at home, like I struggle picking up the washing to hang them out, I struggle to mow the lawn. (Participant 16)

## Issues with self-esteem and intimacy

Nearly all the women reported that rectus diastasis impacted on their confidence, sense of self and that this had ramifications for their social interactions, intimate relationships, and mental health:

You lose a lot of confidence . . . I hated how I looked, visually. Hated it. (Participant 8)

One hundred percent, yes, it’s affected my confidence, just by the sheer bulge that is in front of my tummy. (Participant 23)

It’s horrendous. I still look like I’m 4-months pregnant and it’s horrible. I still get asked all the time if I’m pregnant, it’s totally affected my confidence and my mental health. It’s honestly something that I think about like fifty times a day. Every time I see myself, I see my stomach. (Participant 12)

The majority of the women described some impact on their intimate relationships, more so in how they viewed themselves rather than how their partner viewed them – touching on discursive configurations of the feminine body, ‘standards’ which do not accommodate for the realities of having a child:

I hate him touching me. I’m kind of ashamed of how bad my stomach looks. (Participant 2)

If it comes to the point where I’m having intercourse, like I don’t take my top off or anything, it’s like, ’nope don’t touch my stomach, it’s hideous, it’s been destroyed’. Like it’s a no-go zone for me. (Participant 16)

With my husband? Yes. I don’t feel sexy anymore . . . I think it’s affected my libido . . . I know it’s irrational and I know [my husband] doesn’t care, but I just, that’s not how I see myself, and then I look in the mirror and I remember, and it’s horrible. (Participant 5)

## Barriers to treatment

The women interviewed described a range of barriers to treatment but associated these mostly to a lack of understanding or value by their GP or physiotherapist:

I’ve spoken to several GP’s about it since then and they’ve just all been really dismissive. . . they haven’t given me much information. (Participant 12)

I saw my GP . . . and he just turned around and told me that I need to have surgery or go to a gym. There’s not much that can be done about it. I’ve just got to try and fix it myself, basically. (Participant 9)

Even in instances where women had advocated for themselves, they still faced challenges with being understood and having their needs met by healthcare professionals:

Physios. I’ve lost track of how many physios I’ve seen and not one of them said–nobody ever actually checked my stomach. Yeh, they’d work on my hips and they’d tell me ‘you need to work on your core’ and I said, ‘but I don’t have any core’ . . . no one took that next step of going, ‘maybe it actually has been affected permanently’. (Participant 8)

[The physio] diagnosed me and it was kind of like she didn’t . . . I don’t think she was really familiar with the condition herself . . . like it just seemed like she didn’t really know what to advise me. So, she basically just gave me this booklet which was just pelvic floor exercises for after-birth and she said, ‘just don’t do any sit-ups or anything’. (Participant 14)

Others commented on the limited treatment options available, often reporting an absence of response to conservative treatment or a good response but with limited results. Getting a ‘good response’ could require a significant outlay of time, money, and concentrated energy (up to a year per Participant 19):

I went to a women’s physio . . . I did a Pilates course with her. I don’t feel anything changed. Nothing changed . . . I went to another physio and had like strapping on for a number of weeks . . . nothing works. Still there. Pot belly. Muscle separation. (Participant 1)

I saw a physio early on in the piece, I probably did about 12-months of physio . . . the gap got down to about 3 cm and without surgery it’s not going to join back together and I can’t afford surgery so I’m sort of at the limit of what physio can do to help. (Participant 19)

I’ve discussed it with my GP but nothing further. She sympathises with me. The physio is the same, she says, ‘it’s horrible and it’s annoying but we’ve just got to work with what we can and do the best that we can’. I think that’s what I take out of it. I’ve done pretty much everything that I can do to try to recover it the best I can . . . but it’s still upsetting that this is as good as it gets. (Participant 22)

The barriers to treatment also depended on which treatment option women had engaged with. There were financial and time constraints associated with continuing conservative therapies such as physiotherapy:

You need to be doing the specific exercises every single day to be able to get it to you know a good level. I’ve never been able to do them every single day. (Participant 17)

It’s hard to do stuff mentally and keep on the ball with everything when you’ve got kids and feeding and driving around and overcoming your own personal obstacles . . . You’ve got all these bits of information where . . . you’ve got to make sure you do your exercises. You can’t keep up with that. You just can’t. (Participant 1)

I have to try and get on top of this and start exercising again and then yeh, two kids, and everything else I try and fit in, it just doesn’t really happen. So it perpetuates the cycle. (Participant 14)

Private health is the costly bit . . . once I use all [the rebate for physio] you’re stuck paying nearly 60 per session and that’s what’s costly. (Participant 21)

The first [physio] I saw, she was really expensive’ ‘I went and saw a physio that one of my friends had recommended . . . she was a dedicated women’s physio . . . again she was quite expensive. (Participant 14)

While weekly physiotherapy could cost up to A$3000 per year on these estimates, accessing the surgery was another level up financially:

[Surgery] is very expensive. The first quote was A$25 000, I can’t afford that . . . Why do I have to fork out and put my family in financial stress so I can play with my children? (Participant 2)

I can’t put A$20 000 across for the procedure. . . there’s a lot of money as a barrier. (Participant 1)

I wish [surgery] was cheaper. Honestly, I just think about the expense, even if we get health insurance, it’s going to be so expensive. It feels really unfair. (Participant 5)

## Lack of recognition as a medical condition

Women were asked if they thought ‘post-partum rectus diastasis’ was recognized as a medical condition during interviews. All participants responded that they did not think it was adequately recognized, either by health professionals or society in general:

No, I don’t believe so. Because people don’t understand how debilitating it is unless they’ve gone through it. (Participant 13)

In plastic surgery they are recognising it. I don’t know that it’s too common in GP. (Participant 15)

No [laughs], the fact that the GP had no real idea about it and there’s not a lot out there for people who have it, in terms of treatment options. (Participant 20)

Some women interpreted the lack of Australian Government funding for treatment options as a lack of recognition for the condition – and were astute to the politicized nature of the surgery:

I honestly don’t know, because if it was [recognised as a medical condition], then I suppose that the government would support it a bit more. (Participant 7)

Nope. Not at all. I think if it was then they wouldn’t have taken it off Medicare, to make it possible to get it fixed. (Participant 21)

Women were also asked if they thought surgical repair of rectus diastasis was a cosmetic operation. All women reported that they thought surgery for rectus diastasis repair was a functional or medical procedure and not cosmetic. The degree in which they disagreed with its classification as cosmetic ranged from acknowledging it has some ‘aesthetic’ elements, to being offended by the idea it could be considered cosmetic at all:

No. Not at all. It’s functional. It’s a life-functioning surgery. (Participant 2)

I don’t think it’s considered a cosmetic procedure. I think if you’re muscles aren’t together and that’s how they’re meant to be then that is medical. (Participant 21)

No, God no . . . it’s definitely not cosmetic. It’s a functional procedure. It’s as if someone has done an ACL ligament in their knee and they can’t move around and function and that can be covered, and yet I can’t move around and function with my core but that won’t be covered or won’t get fixed. (Participant 20)

The following participant juxtaposes her reasonable goals – minimizing back pain and getting back some core strength so she can live well – with the perception that the plastic surgeon she saw was focused on cosmetic improvements. In doing so, she showcases that lay populations can misunderstand the medical specialty of plastic surgery and the subtle intersection between its cosmetic and reconstructive elements, such as in abdominoplasty:

I was a little bit miffed that I had to go see a plastic surgeon . . . ‘would you like fries with that?’, ‘would you like a tummy-tuck with that, whilst you’re there?’ I thought my primary goal is to fix this so that I’ve got a bit of core strength, minimise my back pain, and live life to the fullest, but they’re too busy going, ’and we can do a bit of lipo here and do a bit of this’. The focus is all wrong. (Participant 23)

## Overall frustration

All the women who were interviewed expressed feelings of frustration in their experience of rectus diastasis. This related to: the lack of knowledge from health professionals treating the condition; the lack of government funding for surgery, particularly compared to funding for sporting injuries or weight loss surgery; and society’s ignorance of issues affecting post-partum women. Many compared rectus diastasis, which they saw as ‘out of their control’, as more deserving for surgery than massive weight loss patients, who’s obesity they saw as ‘within their control’ as they ‘chose to live an unhealthy lifestyle’:

[It’s frustrating] that the surgery is not claimable on Medicare. It is for weight loss but not when you’ve had kids, which is just ludicrous. (Participant 19)

The disparity in who can have the surgery funded is outrageous. That it’s allowed for people that have been obese and have just lost weight . . . It’s debilitating . . . it’s really affected my quality of life and it’s heart-breaking that the Government doesn’t see it as something that should be fixed and that pregnant women aren’t deserving of it. (Participant 2)

My husband has just recently done his ACL ligament and everyone is really sympathetic to him, yet the same sympathy doesn’t get shared to women that have had children and are still struggling afterwards. (Participant 20)

There should be a lot more help, considering we do go through a hell of a lot even with birth and that and this is what we get afterwards and are just expected to get back on with life and adapt. (Participant 21)

No one seems to feel it’s very important . . . you get this sense that they’re . . . putting across that you’re a bit vain. (Participant 17)

Overall women felt that rectus diastasis was out of their control, and that they felt frustrated at the lack of options to improve their quality of life:

It’s also upsetting . . . if you get to the stage where you’ve done all the physio, you’ve done all the work, like any injury you should be offered surgery and it should be covered under Medicare . . . this isn’t something I went out and planned to do . . . it’s just a consequence of having twins, which also wasn’t something I considered, it just kind of happened. (Participant 22)

After being diagnosed, Participant 1 described the experience as:

Just validating, because I felt that I wasn’t crazy, I was sick of complaining to people.

Similarly, Participant 4 reported after being diagnosed:

I kinda had suspected that anyway, it was nice to have someone confirm it. Rather than it just being, ‘you’re an overtired mum who’s just reading too much into your own body’. It was nice to have someone say, ‘no actually, something is not quite right’. It was actually quite reaffirming.

The two women who had undergone surgery for their rectus diastasis both described significant improvements in their functional ability. One returned to work as a physical education teacher and the other as a flamenco dancer:

If I hadn’t had the surgery, I wouldn’t be able to do my job properly. It’s crazy how much it actually impacts on a person’s life, and unless you’re living it or lived through it, I don’t think a lot of people really understand the impact. (Participant 20)

It should be recognised post-pregnancy . . . you need to get it fixed. Your quality of life is not the same afterwards . . . it’s life changing. You have this confidence that comes back, and I mean, yes you look better, but you actually feel like you’ve got strength back. I can do things that you know I used to be able to do, and then you can’t, and now you can again. When we’re in classes you know, we’re practicing turns and she’ll say use your core to stop you when you’re doing your turns and now it’s like I can actually stop. I can actually do what I’m meant to again. (Participant 8)

## Discussion

Post-partum rectus diastasis is a complex phenomenon. This qualitative analysis reflects the multi-faceted impacts of rectus diastasis on the physical, psychological, and social aspects of women’s lives, as well as how these factors are affected by the healthcare system (and its political economy) with which women engage. The influence of Medicare funding for rectus diastasis surgery, which in Australia is essential for either public hospital treatment or subsidized treatment in the private sector, appeared to impact how these women viewed themselves and their symptoms, as well as their interpretations of how they are treated by healthcare providers and general society. This critically expands upon the findings of Eriksson-Crommert and colleagues, by elucidating how profoundly individuals can absorb the remnants of political decisions regarding medical funding to the point it can impact their lived experience and symptoms.^13^

Due to the unique timing of this analysis, taking place prior to the establishment of the Australian Medicare item number for abdominoplasty for rectus diastasis (which now allows health insurers to contribute majority costs for surgery), the impact of an inconsistent definition of rectus diastasis in the literature could be exposed and appreciated. The inconsistencies in its definition appeared to translate into gaps in the knowledge of healthcare professionals who manage the condition, which impacted the experiences of the women seeking treatment. The themes within our analysis suggest this left women feeling unsupported, dismissed, disillusioned, and frustrated. The limitations or lack of knowledge demonstrated by physiotherapists and GP’s in these women’s accounts, as well as the general misunderstanding of realistic expectations of treatment options in the context of motherhood, reinforced the barriers to treatment experienced by these women, and ultimately translated into a lack of empathy available to the women for genuine suffering.

The introduction of the new Medicare item number for abdominoplasty surgery for rectus diastasis (2022) in Australia may alter how healthcare professionals and women view the condition and its management. We can infer from our research that recognition of the condition as a genuine medical problem is likely to impact a persons’ subjective experience of the condition, in ways that are connected to how easy or difficult it is to access support. Defining the symptoms and criteria objectively via publishing in medical literature and circulating the ideas through medical expertise will help to legitimize individual experiences; illustrated by a contribution of a physiotherapist with some prior knowledge of rectus diastasis within our data: ‘this is not me being lazy, this is a physical condition’. For the women who were unaware of the condition before their diagnosis, an increased recognition of rectus diastasis as a medical condition may have led to an earlier diagnosis and treatment. When it was acknowledged, this realization resulted in feelings of frustration at being dismissed by others, and ‘validation’ at finally being given a diagnosis by healthcare professionals who were knowledgeable in the area. The disparity in the knowledge about post-partum rectus diastasis between healthcare providers was highlighted in the variation between women’s treatment experiences ranging from positive to overtly negative and is a clear area for intervention. It was also apparent that there was some limitation to the conservative treatments accessed, and women felt let down by the Australian health system, even when they had ‘put in the work’ to remediate their muscles. There was an assumed consensus that once they had reached the limits of conservative therapy for their dysfunction, that their functionality was ‘as good as it gets’ (Participant 22). And yet, there is growing evidence that the treatment for rectus diastasis could be improved, such as with surgery or combined physiotherapy and surgery,^6,23^ promising findings that require consolidation, development, and integration into an Australian healthcare system.

The first two themes elicited by this analysis – changed physical appearance and function, and issues with self-esteem and intimacy – appear to be symptoms, signs, and psychosocial impacts that are shared by women universally with post-partum rectus diastasis. These findings align strongly with the findings of Eriksson-Crommert, with women experiencing a permanent physical change to their abdominal wall and its function that greatly lowered their self-esteem and negatively impacted their intimate relationships.^13^ The frequently reported symptoms of abdominal wall weakness, chronic back pain, urinary incontinence, and the impact of post-partum rectus diastasis on women’s work, childrearing, and relationships, as reported by women in our cohort, should form the focus of future quantitative outcomes research on this condition. The objective aspects of these quantitative studies should apply rigorous methodology in order to improve accuracy and reduce bias, such as using ultrasound for diastasis measurement and validated PROMs, as per the recommendations of multiple systematic reviews on rectus diastasis.^12,24,25^

Most of the women in our cohort emphasized the negative physical and mental impacts of rectus diastasis, particularly in reference to caring for young children or engaging in the workforce. Delay to women returning to the workforce due to their physical impairment from childbearing age is likely to compound with long-term impacts on their earning potential, superannuation, and overall financial independence, which is already impacted by having children at all.^26^ Some women interviewed cited that they had significantly changed their work duties or had not been able to return to work, clearly pointing to the experience of rectus diastasis as a barrier to re-entering the workforce – and also compromising the ability of women to seek surgery for the condition privately, which constitutes a double bind for their financial independence and welfare in later life.

Other women identified their back pain was so severe they could not perform routine duties associated with childcare or domestic work, such as picking up children or pushing a pram. However, it is unclear how chronic injuries associated with pregnancy could or should be managed, given that women would not be eligible to return to work programmes that injured workers might be, despite the reproductive capacity of their bodies being seen as a matter of civic duty. In fact, Australia has established pronatalist policies, ideologies, and discourses that encourage women morally, patriotically, and economically to bear children.^27,28^ Perhaps persistent rectus diastasis with significant symptoms that interfere with everyday functioning, child rearing, or return to work should be classified as an impairment or disability, enabling women to access funding from Australian Government organizations such as the National Disability Insurance Scheme.^29^ An alternative option is to fund potentially definitive treatment, such as surgery.

The culmination of symptoms and impact on quality of life elucidated by this qualitative analysis needs to be integrated into the medical definition of rectus diastasis. Once this is established and supported by quantitative studies, there is a need to develop a robust understanding of costs associated with untreated rectus diastasis and its associated impacts on quality of life, childrearing, and workforce engagement, compared with the costs of effective surgical treatment. Some women in this cohort that held private health insurance policies for at least 12 months were able to undergo surgical repair and will hopefully have improvements that reflect the experiences of the two women interviewed who had already undergone surgery. For women without private health insurance (potentially an issue of financial equity), the significant surgical waitlists in the public system will sustain inequitable access.^15,30^

To form a nuanced and responsive definition of post-partum rectus diastasis, we need to allow the qualitative findings presented here to direct and inform future quantitative studies. The changed appearance and function, symptoms of back pain, as well as the psychological and social impacts that capture overall quality of life, should form the focus of these quantitative studies. In conjunction with cost–utility analysis, this will enable the condition to be defined in relation to the social and economic participation of young women as mothers and paid employees, elucidate the multi-layered impacts of rectus diastasis which can unfold for post-partum women, and consolidate appropriate access to surgical treatment within our Australian healthcare system.

## Conclusion

Post-partum rectus diastasis is a multifaceted and complex issue impacting Australian women in a unique way. The themes as reported here reflect how women’s experiences of post-partum rectus diastasis impact their daily lives, social relationships, and engagements with healthcare. The unique timing of our study, situated after the removal and prior to the reinstatement of public funding for abdominoplasty for post-partum rectus diastasis, facilitated an in-depth analysis of how women’s lives and symptoms of the condition are influenced by healthcare availability and medical recognition. This empirically driven exploration of how social structure and patient experience are intertwined is critically innovative and necessary to advance the understanding of patient experience regarding post-partum rectus diastasis.

## Supplemental Material

sj-docx-1-whe-10.1177_17455057241233123 – Supplemental material for Australian women’s experiences of post-partum rectus diastasis: A qualitative study Supplemental material, sj-docx-1-whe-10.1177_17455057241233123 for Australian women’s experiences of post-partum rectus diastasis: A qualitative study by Siobhan Elizabeth Fitzpatrick, Kristen Foley, Tamara Crittenden, David Watson and Nicola R Dean in Women’s Health

sj-xlsx-2-whe-10.1177_17455057241233123 – for Australian women’s experiences of post-partum rectus diastasis: A qualitative study sj-xlsx-2-whe-10.1177_17455057241233123 for Australian women’s experiences of post-partum rectus diastasis: A qualitative study by Siobhan Elizabeth Fitzpatrick, Kristen Foley, Tamara Crittenden, David Watson and Nicola R Dean in Women’s Health This article is distributed under the terms of the Creative Commons Attribution 4.0 License ( http://www.creativecommons.org/licenses/by/4.0/) which permits any use, reproduction and distribution of the work without further permission provided the original work is attributed as specified on the SAGE and Open Access pages ( https://us.sagepub.com/en-us/nam/open-access-at-sage).

## Declarations

**Ethical approval and Consent to participate:** Ethical approval was provided by the Southern Adelaide Human Research Ethics Committee (approval no. 124.21). All participants were provided with a Participant Information Sheet, and informed consent was obtained prior to any participation. Consent to complete the demographic questionnaire was recorded in written format and consent to participate in the interview was recorded audio visually on Zoom immediately prior to the interview as per the ethics requirements.

**Consent for publication:** Informed written consent for publication was provided by all participants.

**Availability of data and materials:** Catalogued and de-identified interview quotes available upon request via email.
