# Supplementary material for: Australian women’s experiences of post-partum rectus diastasis: A qualitative study
Source: Womens Health (Lond). 2024 Apr 5;20:17455057241233123. doi: 10.1177/17455057241233123 (PMC10998484; doi:10.1177/17455057241233123)
Supplement: sj-docx-1-whe-10.1177_17455057241233123 – Supplemental material for Australian women’s experiences of post-partum rectus diastasis: A qualitative study [file sj-docx-1-whe-10.1177_17455057241233123.docx]

Question Guide

*Questions with numerals are the major questions which will be asked during the interview. Sub-questions are prompts to be added based on participant responses. Not all prompt questions will be used and interviewer will be sensitive to any signs of participant distress.*

Thank you for agreeing to participate in this study about rectus diastasis. I’ll be asking you some questions about your diagnosis, associated symptoms and quality of life. If you don’t understand a particular question, or don’t feel comfortable answering it, that is fine, just let me know and we’ll move on. If at any time you feel you need to end the interview, please let me know.

Before we begin, I’d just like to confirm that you are happy to proceed? *[Record affirmation of consent for interview].*

We’ll start with some questions about your diagnosis of rectus diastasis and how it’s impacted you:

1. When and how were you diagnosed with rectus diastasis?

Optional prompts:

1.1 How did you feel after you were diagnosed?

1.2 Did the diagnosis confirm what you already suspected?

1.3 Had you heard of rectus diastasis before?

2. Have you noticed any other symptoms along-side the rectus diastasis?

Optional prompts:

2.1 Do you experience any abdominal pain?

2.2 Any back pain?

2.3 Any change in your ability to hold urine?

2.4 Has your body changed in any other way?

3. Do you think the problem with your abdominal wall has affected how you carry out your normal activities?

Optional prompts:

3.1 Has it limited your ability to work?

3.2 To exercise?

3.3 To socialise?

3.4 If you have other children, does it affect your ability to care for them?

4. Do you think the problem with your abdominal wall has affected your confidence?

Optional prompts:

4.1 Has it affected you in forming relationships?

4.2 Are you in a relationship/If you are in a relationship, do you think that your diastasis impacts on how you feel or act when you are intimate with your partner?

4.3 Do you tend to try to hide your abdomen from your partner?

4.4 Do you avoid looking at your-self in the mirror?

5. Overall, do you think the problem with your abdominal wall has had much of an impact on your quality of life?

Optional prompts:

5.1 Has it affected your mental health?

5.2 Have you sought any care for this?

6. Have you seen any health-care professionals about the condition?

Optional prompts:

6.1 Were they helpful? Were they knowledgeable?

6.2 What treatment options did you discuss?

6.3 Overall, was it a positive or negative experience?

6.4 Are there any barriers to implementing their care-plan?

7. Have you participated in any treatment options?

Optional prompts:

7.1 Were they effective?

7.2 Were they costly?

8. Do you know other women with this condition?

Optional prompts:

8.1 Have they been supportive?

8.2 Are they informative?

8.3 What information resources have you used to learn more about rectus diastasis?

9. Do you think that rectus diastasis is recognised as a medical condition?

Optional prompts:

9.1 Do you think that rectus diastasis repair is recognised as a cosmetic procedure?

9.2 What do you think about the treatment options available?

9.3 If you could change anything about how it is managed, what would it be?

10. Is there anything else you would like to add that we have not covered today?

That is the end of the interview today. Thank you for agreeing to participate in this study. If you have any questions or concerns after you leave, please contact the research contact person, Dr Siobhan Fitzpatrick, whose number or email you can find on the Participant Information Sheet.
